# Supplementary material for: Content-rich biological network constructed by mining PubMed abstracts
Source: BMC Bioinformatics. 2004 Oct 8;5:147. doi: 10.1186/1471-2105-5-147 (PMC528731; doi:10.1186/1471-2105-5-147)
Supplement: Additional File 2 — The original results of the above study (non-essential files are deleted to keep the file size under the limit set by BMC bioinformatics). [file 1471-2105-5-147-S2.bz2 › chilibotAdditionalFile2/dip10/196ID8652557E679/html/ERCC3_ERCC2.html]

 


 **ERCC3** and **ERCC2** 
  
Found 122 abstracts in PubMed, retrieved 10.  
 

 What does Google say? 
 PDF only 
| .edu only 

---

**Interactive relationship** (e.g. stimulation, inhibition, etc)

**Neutral relationship**- Trichothiodystrophy TTD is a rare hereditary multisystem disorder associated with defects in nucleotide excision repair NER as a consequence of mutations in XPD  [ **ERCC2** ] , XPB  [ **ERCC3** ]  or TTDA, three genes that are all related to TFIIH  [ **ERCC3** ] , the multiprotein complex involved in NER and transcription.  Ref: 12393803 Hum Mol Genet, 2002
- We also show that mutations in CSB, as well as in XPB  [ **ERCC3** ]  and XPD  [ **ERCC2** ]  genes, all of which confer CS, disturb the RNA pol I TFIIH  [ **ERCC3** ]  interaction within the CSB IP 150.  Ref: 12419226 Mol Cell, 2002
- S. cerevisiae RAD25  [ **ERCC3** ]  SSL2, RAD3, RAD26, YKU80 HDF2 and RAD54 are the homologue for XPB  [ **ERCC3** ]   **ERCC3** , XPD  [ **ERCC2** ]   **ERCC2** , CSB ERCC6, Ku80 XRCC5 and ATR X HX2, respectively.  Ref: 12638184 Kokuritsu Iyakuhin Shokuhin Eisei Kenkyusho Hokoku, 2002
- General transcription factor IIH TFIIH  [ **ERCC3** ]  consists of nine subunits cyclin dependent kinase 7 Cdk7, cyclin H and MAT1 forming the Cdk activating kinase or CAK complex, the two helicases Xpb  [ **ERCC3** ]  Hay and Xpd  [ **ERCC2** ] , and p34, p44, p52 and p62 refs 1 3.  Ref: 12853965 Nature, 2003
- Moreover, when XPD  [ **ERCC2** ]  mutations prevent interaction with the p44 subunit of TFIIH  [ **ERCC3** ] , transactivation directed by certain nuclear receptors is inhibited, regardless of TTD versus XP phenotype, thus explaining the overlapping symptoms.  Ref: 12820975 Mol Cell, 2003

**Non-interactive relationship** (e.g. studied together, co-existance, homology, etc.)

- However, cells deficient in the other NER genes, XPD  [ **ERCC2** ] , XPB  [ **ERCC3** ]  and XPG, all have the same ratio of exchanges to breaks as wild type.  Ref: 12509251 DNA Repair Amst , 2002
- Here we show that the Drosophila TFIIH  [ **ERCC3** ]  component Xpd  [ **ERCC2** ]  negatively regulates the cell cycle function of Cdk7, the CAK activity.  Ref: 12853965 Nature, 2003
- RESULTS The ERCC1, XPB  [ **ERCC3** ] , and XPD  [ **ERCC2** ]  protein expression patterns yielded significant negative Pearson correlations with 1 7, and 32 out of the 170 compounds, respectively P <.  Ref: 12451985 Ai Zheng, 2002
- In this report, we demonstrate an association of a C terminal domain of human RAD52 amino acids 302 418 with the XPB  [ **ERCC3** ]  and XPD  [ **ERCC2** ]  subunits of transcription factor TFIIH  [ **ERCC3** ]  and RNA polymerase II RNAPII.  Ref: 12372413 Biochem Biophys Res Commun, 2002
- We conclude that the severity of the clinical features in TTD patients and the clinical outcome of differentially mutated XPD  [ **ERCC2** ]  proteins is likely to depend both on the effects that each mutation has on the stability of TFIIH  [ **ERCC3** ]  and on the transcriptional activity of the residual TFIIH  [ **ERCC3** ]  complexes.  Ref: 12393803 Hum Mol Genet, 2002
- We have also measured TFIIH  [ **ERCC3** ]  levels in cells in which different mutations in the XPD  [ **ERCC2** ]  gene are associated with clinical symptoms not of TTD but of the highly cancer prone disorder xeroderma pigmentosum XP.  Ref: 12393803 Hum Mol Genet, 2002
- The antibody study indicated that they thus altered the conformation of one side of TFIIH  [ **ERCC3** ] , consisting of p44, XPD  [ **ERCC2** ] , and Cdk activating kinase subunits, that is essential for the transition stage.  Ref: 12665589 Mol Cell Biol, 2003
- Several NER genes in this system including XPA, XPB  [ **ERCC3** ] , ERCC1, and  **ERCC2**  XPD  [ **ERCC2** ]  have been implicated in anticancer drug resistance in human tumor cells.  Ref: 12451985 Ai Zheng, 2002
